# Tooth Wear Epidemiology and Its Associated Periodontal Health and Sociodemographic Factors in a Cluster of Senior Citizens in Northern Greece

**DOI:** 10.3390/dj10110216

**Published:** 2022-11-18

**Authors:** Charis Theodoridis, George Menexes, Vasiliki Topitsoglou, Sotirios Kalfas

**Affiliations:** 1 Department of Preventive Dentistry, Periodontology, Implant Biology, Faculty of Dentistry, School of Health Sciences, Aristotle University Thessaloniki, 54124 Thessaloniki, Greece; 2 Laboratory of Agronomy, School of Agriculture, Aristotle University of Thessaloniki, 54124 Thessaloniki, Greece

**Keywords:** tooth wear, community periodontal index, periodontal attachment loss, oral hygiene, adults 65–74, periodontal disease epidemiology

## Abstract

Tooth wear (TW) is an irreversible and cumulative phenomenon causing aesthetic and functional compromise. Increasing wear has been associated with age, and various other factors have been reported to influence its type and/or severity both in individuals and groups. Increased TW may constitute a major future problem for the elderly. The present cross-sectional study aims at determining the prevalence of TW in senior citizens from Northern Greece and evaluating the patient-level associations between TW, periodontal condition, and sociodemographic factors. A sample of 363 dentate individuals, aged between 65 and 74 years, was considered according to the WHO guidelines for national pathfinder surveys and three different dentists examined the representative population groups from different urban and rural areas in Northern Greece. The examiners were calibrated prior to the survey, with an interexaminer agreement of over 85%. The simplified TWI, community periodontal index (CPI), attachment loss (AL), plaque index (DI), calculus index (CI), and sociodemographic factors were detected and measured. TW is very prevalent among senior citizens in Northern Greece, with males having been found to experience more wear. Age and oral health status, when measured by the periodontal indices AL, DI, and CI, are significantly combined with TW in both urban and rural areas.

## 1. Introduction

Tooth wear (TW) is an irreversible and cumulative surface loss of mineralized tooth substance due to physical or chemo-physical processes [1], which may lead to functional and aesthetic compromise. It is the combined outcome of dental erosion, attrition, and abrasion and is not considered to be the result of dental caries, resorption, or trauma [1,2].

Although TW should not be attributed only to the aging process, a general tendency towards increasing wear depending on age has been supported. [3,4]. This may be particularly significant nowadays, regarding increased life expectancy [5], as well as preventive dentistry considerations, and dental restorative innovation procedures might enhance the possibility of natural dentition retention among elderly populations. More specifically, a decrease in edentulousness and an increase in remaining teeth have been reported with regard to senior European citizens [6,7], indicating that more teeth and tooth surfaces are at risk of disease or TW and, consequently, that this phenomenon may constitute significant pathological manifestations, such as severe sensitivity and pulp inflammation [8]. As a result, the growing concern regarding maintaining oral health in the elderly, as well as the potential impact of advanced TW lesions on both treatment planning and patients’ quality of life, has intensified the interest in epidemiological data concerning TW in such populations [9,10]. A recent study by our department indicates that senior citizens in Northern Greece may present high values of TW and that TW severity may be exacerbated even more with increasing age. Because of the relatively small sample size in our first study, which was limited to 70 urban participants in a single elderly people’s day center (EPDC), and due to the different study design, which was more for analyzing the presence and the extent of severe wear rather than providing a cumulative TW analysis concerning different sociodemographic aspects in community subpopulations, we argued that the further investigation of TW in older Greeks might be of interest [11].

Except for age, various contributing factors have been reported to affect the prevalence and severity of mineralized tooth substance loss, with factors affecting attrition (e.g., bruxism), erosion (e.g., acidic dietary habits) and abrasion (e.g., excessive toothbrushing) being the most well-analyzed factors. Sociodemographic parameters, such as gender or urban/rural population, were connected with different levels or TW in Chinese adults aged over 40 years [12], while a tendency for low socioeconomic participants to present higher mean tooth wear scores was monitored in the age cluster of 65–74 years in the Netherlands as well as in a more recent cross-sectional study of the Chinese adult population [4,13]. From a periodontal perspective, little evidence has been reported so far with regard to probable associations between periodontal status and TW. Early findings from studies evaluating tooth brushing and its effect on cervical wear, suggest that specific brushing techniques may enhance abrasion and hence provoke cervical wear [14,15]. Clinical periodontal parameters, such as plaque index, probing pocket depth, gingival recession, and tooth mobility were assessed as influential factors on TW in 30 adult patients in a more recent study [16], which detected cervical wear to be significantly associated with less plaque accumulation and more shallow periodontal pockets. On the other hand, advanced gingival recession with an absence of tooth mobility predisposes a person to the development of deeper cervical lesions. Nevertheless, cumulative tooth levels or patient level TW, and not just the cervical component of it, has yet to be investigated with regard to periodontal status in large community samples.

In the scope of the above, the aims of the present cross-sectional study were to investigate the prevalence of TW in a large sample of Northern Greek senior citizens and to evaluate the patient-level associations between TW and periodontal condition, as well as the main sociodemographic factors and behavioral parameters.

## 2. Materials and Methods

A cluster sample was selected according to the WHO guidelines for national pathfinder surveys. This design ensures the participation of a satisfactory size of people that may present different disease prevalences for the conditions that are being examined [17]. In order to make a comparison with previous national survey data and to be more accurate, the sample was collected in the same manner and from the same geographical areas of Northern Greece as in past national epidemiological studies [18,19,20]. More specifically, the study enrolled samples from the metropolitan city of Thessaloniki, as well as from four Northern Greece counties (Evros, Ioannina, Kastoria, and Larissa). Three communities of differing socioeconomic backgrounds were selected randomly in Thessaloniki, while one urban and one rural community were selected randomly within each of the aforementioned counties, resulting in 11 different site samples. Senior citizens (age: 65–74 years) were selected and examined according to the WHO national pathfinder survey methodology for this particular age group [17]. Permission from the Greek Ministry of Health (#143207/15-12-2012) was obtained before the clinical examinations. The Ethical Committee of Aristotle University, Thessaloniki, approved the present cross-sectional study as well, with regard to data processing and analysis (#11/01-07-2020).

The final sample comprised 363 dentate individuals in total, who were examined by three dentist examiners, supported by their assistants. The examinations were carried out under artificial light (daray lamps) using dental mirrors and the WHO CPI periodontal probe. Cotton rolls and gauze were available for moisture control and biofilm removal when necessary. Prior to the survey beginning, examiners were trained and calibrated on the examined indices, with very good interexaminer agreement (Kappa Coefficient > 0.85).

The primary recorded variable was the tooth wear index (TWI) [21]; more specifically, a simplified version of the original Smith & Knight TWI [22], which requires measurements of 40 tooth surfaces from 16 teeth. Because of the absence of cervical surfaces among those being examined via the simplified TWI, cervical surfaces of the same 16 teeth were screened and included within the overall TWI, which was expressed as mean value per patient. Periodontal health was recorded using the community periodontal index (CPI) and presented as mean values per patient, as well as with an ordinal version of attachment loss (AL) introduced by WHO [17]. Simplified oral hygiene index [23] was utilized in order plaque (DI) and calculus (CI) to be assessed, and the scores were classified into three levels, as described by Greene [24]. Sociodemographic data, including gender, age, area, urban/rural location, educational level, and monthly income, were collected through a structured questionnaire that was completed face-to-face at the time of the clinical examination. Further information concerning these periodontal health indices and the investigated sociodemographic factors are provided in our recent publication [25]. Furthermore, participants were asked about some behavioral parameters and, more specifically, whether they feel any pain/discomfort during cold food/drinks uptake (Q1), whether they brush their teeth immediately after food/drink uptake (Q2), whether they grind or clench their teeth unconsciously (Q3), and whether they bite or clench any hard items with their teeth (Q4).

The data were summarized by calculating the absolute and relative frequencies (percentages %), indices of central tendency (mean and median values), and indices of variability (minimum and maximum values, standard deviations, and standard errors of mean values). All the study indices, sociodemographic factors, and questions Q1–Q4 were utilized and analyzed on a participant-level basis.

Continuous variables were compared among participants’ various groupings using a Kruskal–Wallis (KW) or a Mann–Whitney (MW) test, as appropriate. In all hypotheses and testing procedures (MW-tests and KW-tests), the observed significance level (*p*-value) was computed with the Monte-Carlo simulation method based on 10,000 resampling circles [26]. This simulation method leads to valid inferential conclusions even in cases where the methodological presuppositions of the statistical test used are not satisfied (e.g., random samples, independent observations, symmetrical distributions, and the absence of “heavy” outliers).

All statistical analyses were performed with the IBM SPSS v.23.0 enhanced with the module Exact Tests (for performing the Monte-Carlo simulation method) [26]. The significance level in all hypothesis and testing procedures was predetermined at *a* = 0.05 (*p* ≤ 0.05).

## 3. Results

Of the 363 Greek senior citizens who were examined and participated, 188 (51.8%) were males and 175 (48.2%) were females. A total of 229 (63.1%) individuals were urban inhabitants, whereas 135 (36.9 %) resided in the rural areas that were enrolled. The descriptive statistics (central tendency and dispersion) for TWI and the periodontal health indices (DI, CI, CPI, and AL) are presented in Table 1. A statistically significant medium positive correlation was detected between TWI and all the periodontal health indices eecept CPI. The Spearman’s rho coefficient for DI was 0.25 (*p* < 0.001), while the corresponding coefficients for CI and AL were 0.25 (*p* < 0.001) and 0.21 (*p* < 0.001), respectively. The Spearman’s rho correlation coefficient between TWI and age was 0.19 (*p* < 0.001), indicating a weak positive correlation between TWI and the specific individual ages within the examined age cluster.

The results for the presence of pain/discomfort during cold drinks or food uptake (Q1), tooth brushing immediately after food/drink uptake (Q2), unconscious teeth grinding or clenching (Q3), and hard object biting or clenching (Q4) are presented in Table 2. The descriptive statistics for TWI concerning the sociodemographic factors and questions Q1–Q4 are presented in Table 3.

The MW test indicated a statistically significant association between TWI and gender (*U* = 13,628, *Z* = −2.825, and *p* = 0.005), although TWI was not found to be significantly different with regard to urban and rural inhabitants (*U* = 14,004.5, *Z* = −1.388, and *p* = 0.165). The KW test indicated significant differences among the examined regions relative to TWI (*X*^2^ = 18.21, df = 4, and *p* = 0.001). On the other hand, no significant association was found either between the TWI and the level of education (KW test, *X*^2^ = 6.99, df = 5, and *p* = 0.228) or between TWI and monthly income (ΚW test, *X*^2^ = 0.59, and df = 2, *p* = 0.757). The TWI scores within the Q1 responses (MW test, *U* = 10,133, *Z* = −1.914) remained marginally insignificant (*p* = 0.056). Nevertheless, a statistically significant difference in TWI was revealed between the Q2 responses (MW test, *U* = 12,156, *Z* = −3.377, and *p* < 0.001) and between the Q3 responses (MW test, *U* = 8316, *Z* = −2.598, and *p* = 0.010), while the TWI scores did not show any significant difference between the Q4 responses (MW test, *U* = 6360, *Z* = −0.286, and *p* = 0.766).

## 4. Discussion

The presence of TW in senior citizens from Northern Greece appears to be almost universal. In fact, only 2 out of the 363 study participants presented zero TWI average scores, indicating that few of the tooth surfaces were regarded as intact, without any degree of detected wear. Although the published data on the presence of TW in adult populations are less common than for children or adolescents (primarily because of recruitment factors [27]), this finding stands in accord with our previous study [11] as well as with a similar finding of 100% prevalence of wear in Chinese adults aged 50 to 74 years [4]. Another recent cross-sectional study stands in line with these TW prevalence levels, reporting a 97.9% TW presence in Chilean participants [28]. For all of its high prevalence, TW in the elderly is characterized by a wide range of severity, varying from partial enamel loss to pulp exposure and crown elimination. Apparently, the vast majority of the worn sites may not be considered as diseased or pathological, especially in older adults; nevertheless, in specific individuals presenting increased wear, such as dentine/pulp exposure or occlusal wear, which may lead to vertical dimension compromise, or with symptoms compatible with TW, such as dentine hypersensitivity, therapeutical interventions may be considered on condition that the underlying risk factors can be addressed.

To the best of our knowledge, this is the first cross-sectional study that investigates TW in the age cluster of senior citizens (65–74 years) in a Mediterranean and/or Balkan country. It is also the only epidemiological study that attempts to investigate the relationship between TW and periodontal status on a patient-level basis after having enrolled a large sample of specific age group participants. While a statistically significant positive correlation between attachment loss and the TWI scores was found, when CPI was examined, significance was not detected. The exact definition of these periodontal indices, both being utilized as ordinal variables, may interpret this outcome. Consequently, it can be advocated that, in individuals with more advanced forms of periodontal disease, presenting compromised periodontium and loss of attachment, TW may be more likely to occur, especially when gingival recession is present since the revealed root surfaces are more susceptible to abrasion forces. On the other hand, a positive CPI value does not necessarily suggest either the presence of periodontitis or gingival recession in the examined sites, but it may indicate gingival bleeding (CPI = 1), calculus presence (CPI = 2) or shallow/medium periodontal pocketing (CPI = 3); when the CPI score is greater than 3 in a specific site, loss of attachment is also expected to be found, and the AL index is expected to be also positive. Furthermore, higher TW in participants presenting with positive AL may also be interpreted as a characteristic of a more general tendency of some people towards dental negligence, indicating a lack of oral health culture and absence of dental care. This hypothesis is compatible with the significant positive association between TW and the oral hygiene indices (DI and CI), which was also detected in our study. When considering these relationships from the opposite direction, it may be assumed that TW lesions, especially on cervical surfaces, may predispose a person to periodontal inflammation initiation or recurrence by creating surfaces more prone to biofilm accumulation and are harder to reach through toothbrushing techniques (cervical grooves, V-shaped defects, crevices, etc.).

A positive correlation between TWI and the specific individual ages of the examined participants was revealed, even though the subjects belonged to the same age group (age: 65–74 years). This association emphasizes the role of aging towards TW evolution since the latter is considered a common experience and, for most, an almost universal outcome of aging {D Bartlett and S O’Toole, 2019}. TWI analyses, with regard to other sociodemographic factors, revealed a significance referring to gender, with the TWI scores being higher in males than in females. Male gender has also previously been detected as a factor significantly related to TW in European adult populations [13,29,30]. Although no definite conclusions should be drawn concerning the reason why men show more tooth wear, it has been hypothesized that this may be attributed to the fact that men consume more acidic drinks than females, and the fact that their masticatory muscles exert higher forces [13]. Socioeconomic status (monitored by level of education and income) did not show any significant association with TW in the present study; this has been stated in numerous past studies in the literature [31,32]. However, this is not regarded as a universal finding since there have been reported data indicating that lower socioeconomic status may be combined with higher TW scores [4,13].

About one out of four (24%) of the examined subjects reported pain/discomfort during cold food/drink uptake (Q1), while about 40% of the participants reported toothbrushing immediately after food/drink uptake (Q2), with the latter being significantly correlated with TWI. This may be attributed to the fact that toothbrushing immediately after food/drink uptake might lead to an increased risk of wear due to a combination of abrasion (mechanical wear) against a background of recent erosion (chemical wear) by probable acidic substance consumption. The average TWI score of the participants was also associated with unconscious tooth grinding or clenching, which is indicative of the attrition component of the tooth wear phenomenon. Mechanical stress loading might be compatible with TW lesions, especially on occlusal/incisal surfaces, and the positive bruxism response has been associated with dentine exposure in a large sample of Chinese adults [4]. This finding is consistent with the empirical belief among clinicians that TW becomes more severe during parafunctional activities, such as bruxism [33], as well as with recent evidence concerning TW and bruxism association [34].

## 5. Conclusions

The results of this cross-sectional study are consistent with the universal prevalence of tooth wear in senior citizens aged 65–74 years.

A significant difference in the tooth wear index scores was detected, referring to sociodemographic factors, such as the age and gender of the participants. Comparably, a significant association between the index and pain during cold diet uptake and unconscious tooth grinding or clenching was revealed.

Periodontal status, expressed in terms of loss of attachment, as well as the presence of plaque and calculus, were significantly associated with tooth wear in the examined individuals.

## Figures and Tables

**Table 1:** Descriptive statistics for Tooth Wear Index and periodontal health indices.

Index	Minimum	Median	Maximum	Mean	Std. Deviation	Std. Error of Mean
DI average	0	1.00	3.00	1.30	0.79	0.04
CI average	0	1.33	3.00	1.45	0.85	0.05
CPI average	0	2.50	4.00	2.39	0.78	0.04
AL average	0	0.83	7.00	0.94	0.79	0.04
TWI average	0	0.88	4.00	0.96	0.53	0.03

Plaque index (DI), calculus index (CI), Community Periodontal Index (CPI), and Attachment Loss (AL), Tooth Wear Index (TWI).

**Table 2:** Frequency of the participants answers for questions Q1–Q4.

Q1: Do you feel any pain/discomfort during cold food/drinks uptake?		
	Frequency	Percent (%)
No	273	76
Yes	86	24
Total	359	100
Q2: Do you brush your teeth immediately after food/drink uptake?		
	Frequency	Percent (%)
No	220	61.3
Yes	139	38.7
Total	359	100
Q3: Do you grind or clench your teeth unconsciously?		
	Frequency	Percent (%)
No	288	80
Yes	72	20
Total	360	100
Q4: Do you bite or clench any hard items, with your teeth?		
	Frequency	Percent (%)
No	319	88.6
Yes	41	11.4
Total	360	100

**Table 3:** TWI descriptive statistics for the variables that underwent analysis.

	Minimum	Median	Maximum	Mean	Std. Deviation	Std. Error of Mean	*n*
Gender							
Males	0	1.00	4.00	1.02	0.56	0.04	188
Females	0	0.80	2.83	0.89	0.50	0.04	175
Total	0	0.88	4.00	0.96	0.53	0.03	363
Population types							
Urban	0	0.95	4.00	1.02	0.56	0.04	229
Rural	0	0.82	2.83	0.89	0.50	0.04	134
Total	0	0.88	4.00	0.96	0.53	0.03	363
Region							
Thessaloniki	0	0.95	3.53	1.02	0.59	0.06	105
Ioannina	0	1.00	2.83	1.02	0.53	0.07	64
Larissa	0	1.02	2.00	0.99	0.49	0.06	65
Kastoria	0	0.98	1.98	0.95	0.40	0.05	64
Evros	0.17	0.66	4.00	0.80	0.59	0.07	65
Total	0	0.88	4.00	0.96	0.53	0.03	363
Monthly Income							
≤590 €	0	0.87	3.00	0.95	0.50	0.04	162
591–1200 €	0	0.91	3.57	0.96	0.53	0.04	174
>1200 €	0.17	0.82	1.67	0.86	0.42	0.08	25
Total	0	0.88	3.53	0.95	0.51	0.03	361
Level of education							
Illiterates	0	0.91	2.00	0.91	0.54	0.10	31
Basic	0	0.89	4.00	0.96	0.51	0.03	231
Lower secondary	0	1.00	3.53	1.14	0.69	0.10	40
Secondary graduates	0	0.84	1.95	0.86	0.48	0.09	30
Nonuniversity tertiary	0.25	0.72	2.50	0.88	0.55	0.15	14
University graduates	0.17	0.67	1.61	0.76	0.42	0.10	16
Total	0	0.88	4.00	0.96	0.53	0.03	362
Q1							
No	0	0.94	4.00	0.99	0.56	0.03	273
Yes	0	0.81	1.98	0.86	0.43	0.05	86
Total	0	0.88	4.00	0.96	0.53	0.03	359
Q2							
No	0	1.00	4.00	1.02	0.53	0.04	220
Yes	0	0.75	3.53	0.87	0.53	0.04	139
Total	0	0.88	4.00	0.96	0.53	0.03	359
Q3							
No	0	0.86	4.00	0.93	0.54	0.03	288
Yes	0.18	1.08	2.25	1.07	0.48	0.06	72
Total	0	0.89	4.00	0.96	0.53	0.03	360
Q4							
No	0	0.90	4.00	0.97	0.54	0.03	319
Yes	0.23	0.82	2.83	0.95	0.51	0.08	41
Total	0	0.89	4.00	0.96	0.53	0.03	360

## Data Availability

The datasets used and/or analyzed during the current study are available from the corresponding author on reasonable request.
